# Supplementary material for: Systematic Review of the Survival Outcomes of Neoadjuvant Chemotherapy in Women with Malignant Ovarian Germ Cell Tumors
Source: Cancers (Basel). 2023 Sep 8;15(18):4470. doi: 10.3390/cancers15184470 (PMC10526733; doi:10.3390/cancers15184470)
Supplement: Supplementary file 1 [file cancers-15-04470-s001.zip › cancers-2573468-supplementary.pdf]

Supplemental File S1. Search keywords.

PubMed

#1 Ovarian Neoplasms [MeSH]
#2 Pelvic Neoplasms [MeSH]
#3 Fallopian Tube Neoplasms [MeSH]
#4 Adnexal Diseases [MeSH]
#5 ovary [tiab] OR ovaries [tiab] OR ovarian [tiab] OR adnexa [tiab] OR fallopian [tiab] OR peritoneal [tiab]
#6 neoplasm [tiab] OR cancer [tiab] OR cancers [tiab] OR carcinoma [tiab] OR carcinomas [tiab] OR malignan* [tiab] OR tumor* [tiab] OR tumour* [tiab]
#7 #5 AND #6
#8 #1 OR #2 OR #3 OR #4 OR #7
#9 Neoadjuvant therapy [MeSH]
#10 Neoadjuvant [tiab] OR "followed by interval debulking" [tiab] OR "followed by cytoreduc*" [tiab] OR "primary chemotherapy" [tiab]
#11 #9 OR #10
#12 #8 AND #11
#13 Neoplasms, Germ Cell and Embryonal [MeSH] OR "germ cell" OR GCT OR Non-epithelial OR Dysgerminoma OR Immature teratoma OR yolk sac
#14 #12 AND #13

Scopus

#1 TITLE-ABS-KEY(ovarian OR ovary OR ovaries OR adnexa OR peritoneal OR fallopian) W/2 (neoplasm OR cancer OR malignan* OR tumor OR tumour OR carcinoma)
#2 Neoadjuvant OR "followed by cytoreductive surgery" OR "followed by interval debulking" OR "primary chemotherapy" OR "before surgery"
#3 #1 AND #2
#4 "germ cell" OR GCT OR Non-epithelial OR Dysgerminoma OR Immature teratoma OR yolk sac
#5 #3 AND #4

Cochrane

#1 MeSH descriptor: [Ovarian Neoplasms]
#2 MeSH descriptor: [Adnexal Diseases]
#3 MeSH descriptor: [Fallopian Tube Neoplasms]
#4 MeSH descriptor: [Pelvic Neoplasms]
#5 #1 OR #2 OR #3 OR #4
#6 (germ cell or non-epithelial or dysgerminoma or immature teratoma or yolk sac) near/3 (cancer* or carcinoma* or malignan* or mass or masses or cyst or cysts or neoplasm* or tumor* or tumour*)

#7 (non-epithelial) near/5 (ovar*)
#8 #6 OR #7
#9 #5 AND #8
#10 MeSH descriptor: Neoadjuvant Therapy
#11 #9 AND #10

Web of Science
#1 ovary OR ovaries OR ovarian OR adnexa OR fallopian OR peritoneal
#2 neoplasm OR cancer OR cancers OR carcinoma OR carcinomas OR malignan* OR tumor* OR tumour*
#3 #1 AND #2
#4 Neoadjuvant OR "followed by interval debulking" OR "followed by cytoreduc*" OR "primary chemotherapy"
#5 #3 AND #4
#6 Neoplasms, Germ Cell and Embryonal OR "germ cell" OR GCT OR Non-epithelial OR Dysgerminoma OR Immature teratoma OR yolk sac
#7 #5 AND #6

Supplemental File S2. Metadata of eligible studies.

Author	Year	Location	Type	Total	Stage	II-IV	NACT	Rate (all)	Rate (adv)	Hist	Outcomes
Agrawal A[1]	2023	IND	Retro	31	I-IV	--	2	6.5%	--	Dys, MG	--
Newton C[2]	2019	GBR	Retro	138	I-IV	49	16	11.6%	32.7%	Dys, MG, IT, YST, PNET	OS, EFS
Agarwal R[3]	2019	IND	Retro	48	I-IV	17	11	22.9%	64.7%	Dys, MG, IT, YST	RR, OS, DFS
Divya S[4]	2019	IND	Retro	10	--	--	2	25%	--	EC	--
Lakshmanan M[5]	2018	IND	Retro	38	I-IV	33	27	71.1%	81.8%	Dys, Ter, MG, YST, CHO	RR, OS, DFS
Zhang GY[6]	2018	CHN	Retro	18	IIIC-IV	18	18	100%	100%	YST	OS, DFS
Calzas RJ[11]	2016	SPN	Case	1	III	1	1	--	--	Dys	--
Amirthalingam V[12]	2016	IND	Case	1	III	1	1	--	--	MG	--
Puangthong U[13]	2015	THA	Case	1	--	1	1	--	--	MG	--
Lu Y[7]	2014	CHN	Retro	53	III-IV	53	21	39.6%	39.6%	YST	Rec, PFS
Talukdar S[8]	2014	IND	Retro	66	III-IV	23	23	34.8%	34.8%	Dys, MG, YST	OS, DFS
Raveendran A[14]	2010	IND	Case	2	I-III	--	2	100%	100%	MG, Dys	--
Bafna UD[9]	2001	IND	Retro	33	IA-IV	--	4	12.1%	--	Dys, non-Dys	RR
Baranzelli MC[10]	2000	FRN	Retro	49	I-IV	48	12	24.5%	25%	Non-seminoma	RR, OS
Kawai M[15]	1999	JPN	Case	1	Ia	0	1	100%	100%	YST	DFS, OS

Some values are estimated by the authors. Abbreviations: IND, India; GBR; United Kingdom; CHN, China; SPN, Spain; THA, Thailand; FRN, France; JPN, Japan; Type, type of study; Total, total number of included cases, II-IV, number of women with stage II-IV; NACT, number of women who had neoadjuvant chemotherapy; rate (all), women with NACT/ total number of women ; rate (adv), women with NACT/ number of women with advanced disease (stage II-IV); Rec, rate of recurrence; RR, response rate; Hist, histology; outcomes; reported outcomes; Dys, dysgerminoma; MG, mixed germ cell tumor; IT, immature teratoma; EC, embryonal carcinoma; MT, mature teratoma; TER, teratoma; PNET, primitive neuroectodermal tumor; CHO, Choriocarcinoma; YST, yolk sac tumor; Retro, retrospective study; Case, case report or case series.

Supplemental Table S3. Risk of bias assessment for the comparator study.

Authors	Confounding	Selection	Classification of intervention	Deviations from interventions	Missing data	Measurement of outcomes	Reported results	Overall bias
Agarwal R[3]	● (serious)	● (serious)	● (moderate)	● (no information)	● (no information)	● (moderate)	● (moderate)	● (serious)
Lakshmanan M[5]	● (serious)	● (serious)	● (moderate)	● (no information)	● (no information)	● (moderate)	● (serious)	● (serious)
Lu Y[7]	● (serious)	● (moderate)	● (moderate)	● (no information)	● (no information)	● (serious)	● (moderate)	● (serious)
Talukdar S[8]	● (serious)	● (moderate)	● (moderate)	● (no information)	● (no information)	● (moderate)	● (moderate)	● (serious)

Risk of bias assessment was performed using the Risk Of Bias In Non-randomized Studies–of Interventions tool (ROBINS-I) [4-6].

● Low risk of bias (the study is comparable to a well-performed randomized trial with regard to this domain)

● Moderate risk of bias (the study is sound for a non-randomized study with regard to this domain but cannot be considered comparable to a well-performed randomized trial)

● Serious risk of bias (the study has some important problems in this domain)

● Critical risk of bias (the study is too problematic in this domain to provide any useful evidence on the effects of intervention.

● No information on how to base a judgment on the risk of bias for this domain.

References

1. Agrawal A, Podder AR. Neoadjuvant Chemotherapy Versus Maximal Cytoreduction for Malignant Germ Cell Tumors of the Ovary. Indian Journal of Gynecologic Oncology 2022;21:10.
2. Newton C, Murali K, Ahmad A, et al. A multicentre retrospective cohort study of ovarian germ cell tumours: Evidence for chemotherapy de-escalation and alignment of paediatric and adult practice. European journal of cancer (Oxford, England : 1990) 2019;113:19-27.
3. Agarwal R, Rajanbabu A, Keechilattu P, Nair IR, Vijaykumar DK, Unnikrishnan UG. A retrospective analysis of the pattern of care and survival in patients with malignant ovarian germ cell tumors. South Asian journal of cancer 2019;8:35-40.
4. Divya. S, Syamala O, Rani GU, et al. Malignant Ovarian Tumors in Adolescents: A Case Series. J South Asian Feder Obst Gynae 2019;11(5):331–335.
5. Lakshmanan M, Gupta S, Kumar V, et al. Germ Cell Tumor Ovary: an Institutional Experience of Treatment and Survival Outcomes. Indian journal of surgical oncology 2018;9:215-19.
6. Zhang GY, Zhang R, Wu LY, Li B, Li SM. Neoadjuvant Bleomycin, Etoposide, and Cisplatin (BEP) Chemotherapy in the Treatment of Extensively Advanced Yolk Sac Tumors: A Single Center Experience. International journal of gynecological cancer : official journal of the International Gynecological Cancer Society 2018;28:713-20.
7. Lu Y, Yang J, Cao D, et al. Role of neoadjuvant chemotherapy in the management of advanced ovarian yolk sac tumor. Gynecologic oncology 2014;134:78-83.
8. Talukdar S, Kumar S, Bhatla N, Mathur S, Thulkar S, Kumar L. Neo-adjuvant chemotherapy in the treatment of advanced malignant germ cell tumors of ovary. Gynecologic oncology 2014;132:28-32.
9. Bafna UD, Umadevi K, Kumaran C, Nagarathna DS, Shashikala P, Tanseem R. Germ cell tumors of the ovary: is there a role for aggressive cytoreductive surgery for nondysgerminomatous tumors? International journal of gynecological cancer : official journal of the International Gynecological Cancer Society 2001;11:300-4.
10. Baranzelli MC, Bouffet E, Quintana E, Portas M, Thyss A, Patte C. Non-seminomatous ovarian germ cell tumours in children. European journal of cancer (Oxford, England : 1990) 2000;36:376-83.
11. Calzas Rodríguez J, Carmen Juarez Morales MD, Casero MA. Death by bleomycin pulmonary toxicity in ovarian dysgerminoma with pathologic complete response to chemotherapy. A case report. Respiratory medicine case reports 2016;18:48-50.
12. Amirthalingam V, Sharma TD, Rai PC, Singh TT, Devi KP. Malignant mixed germ cell tumor of ovary presenting as advanced disease in an adolescent girl. European journal of gynaecological oncology 2016;37:750-52.
13. Puangthong U, Pongpirul K. Chemotherapy-induced acute psychosis in a patient with malignant germ cell tumour. BMJ case reports 2015;2015.
14. Raveendran A, Gupta S, Bagga R, et al. Advanced germ cell malignancies of the ovary: should neo-adjuvant chemotherapy be the first line of treatment? Journal of obstetrics and gynaecology : the journal of the Institute of Obstetrics and Gynaecology 2010;30:53-5.
15. Kawai M, Ohno Y, Shibata D, et al. Primary chemotherapy in strongly suspected yolk sac tumor of the ovary. International Journal of Clinical Oncology 1999;4:48-51.
